# Hyperglycemic effects on limbs tingling and twisting sensations

**DOI:** 10.3389/fneur.2025.1591279

**Published:** 2026-01-05

**Authors:** Samaa Faez Khudhur

**Affiliations:** 1 Faculty of Science, University of Thi-Qar, Nasiriya, Iraq; 2 Department of Pathological Analysis, College of Science, University of Thi-Qar, Nasiriya, Iraq

**Keywords:** numbness, limbs cramping, higher blood sugar, nerve damage, peripheral neuropathy, carpal tunnel

## Abstract

What Hyperglycemia Does to the Legs is quite devastating and ends with the tragic event of losing lower limbs. A patient with diabetic neuropathy (DN) may have both good and sensory loss. On one hand, there are symptoms like pain, sensitivity, tingling, cramping, and cold feet. On the other hand, there are negative symptoms, including sensory loss and delayed wound healing the cues back up this negative out comes (Sensory nerves are essential for healing wounds because they help regulate the immune system and encourage the movement of cells, the creation of growth factors, and the reproduction of cells). When sensory nerves are injured or cut off, there is a decline in neuropeptides and white blood cell presence, which slows down the healing process. This problem is especially seen in diabetic neuropathy, as the loss of sensory functions greatly affects the ability to heal wounds, Chronic wounds display high levels of inflammation, reduced activity of growth factors, and poor oxygen supply to tissues, which all lead to slow healing and the persistence of non-healing wounds. The development and progression of DN cannot be explained solely by elevated blood glucose levels. New evidence suggests that a naturally occurring reactive metabolite called MG (methylglyoxal) can enhance function by modifying neuronal ion channels involved in chemo sensing and action potential generation in nociceptive nerve endings. This modification occurs after Glo1 (glyoxalase I) levels are reduced. One possible reason for the development of DN is the effect of dicarbonyls on the neuronal compartment. Thus, there may be new and improved ways to treat DN if we focus on preventing the buildup and effects of MG.

## Introduction

Diabetes can cause a type of nerve damage known as diabetic neuropathy, which can manifest in any person who has diabetes. The presence of high blood sugar (glucose) was found to promote nerve damage throughout the entire body (1). The most common nerves that were impacted were those that were located in the legs and feet. One way to determine the amount to which a diabetic patient’s body is affected is to determine the degree of nervosa damage (2). The symptoms of DN include discomfort and numbness in the legs and feet, as well as problems with the internal gastrointestinal tract, blood vessels, urinary tract, and heart (3, 4). Distal symmetric neuropathy is the most prevalent form of distal neuropathy, accounting for approximately 75% of all occurrences of the condition. Set-up neuropathy is a condition that can occur in diabetic people who have an asymmetric etiology. That, in the past, were brought on by metabolic changes and ischemia, but are today brought on by immunological alterations (3): clear interpretation as following, 1- Uneven in character: This indicates that the nerve injury is impacting one side of the body more than the other, typically beginning in a particular spot rather than being balanced on both sides. 2-Causes: This term describes what leads to or brings about a disease. This kind of neuropathy in diabetic individuals was believed to primarily result from: A-Changes in metabolism: Fluctuations in blood sugar can harm nerves directly. B- Lack of oxygen: Decreased blood circulation (caused by injured blood vessels from diabetes) can starve nerves of essential oxygen and nutrients, resulting in injury (5). The objective sensory testing, nerve conduction analysis, and autonomic testing are the two of these five procedures that are recommended for use in the injury [laboratory examination of symptoms and signs associated with developmental neuropathy (DN)]. Lipoic acid Alpha-lipoic acid (ALA), known for its strong antioxidant effects, has several benefits: 1-It lessens oxidative stress from high blood sugar levels, which can harm nerves and lead to nerve pain.2-It boosts nerve performance by increasing the speed of nerve signals and aiding in the repair of nerve fibers. 3-It has properties that fight inflammation, helping to lower neuroinflammation and reduce pain. 4-It plays a role in regulating metabolism by enhancing how glucose is processed, lowering protein glycation, and raising insulin sensitivity. 5-Its ability to relieve pain includes antioxidant functions and decreasing the activity of pain receptors associated with nerve pain signals (6, 7) and L-carnitine, especially in its form as acetyl-L-carnitine (ALC), is made naturally in our bodies and turns food into energy. -Promotes the health and repair of nerve cells.-Has been proven to help with nerve pain and enhance the sense of vibration in people with diabetes. -Provides relief from nerve pain, particularly when treatment starts early for diabetic nerve issues. -Helps nerve fibers to regenerate and lessens symptoms such as pain, while avoiding common side effects of other treatments (8, 9), both of which are non-steroidal medications, should be provided to patients who are experiencing unpleasant neuropathic pain, analgesics, anti-inflammatory, antidepressant, and anticonvulsant symptoms in order to regulate hyperglycemia, which is a risk factor for cardiovascular disease,. On the other hand, all diabetic people experience consequences such as neuropathy, in spite of the fact that this condition can be avoided or slowed down if blood sugar levels are regulated and a healthy diet is followed, the prevalence of this ailment continues to rise. Leukocytes, which are white blood cells, as well as other cells in the body, such as helper T cells of the second type Th2, are responsible for the secretion of interleukin-10, Leukocytes, including helper T cells such as Th2 cells, secrete interleukin-10 (IL-10), which is an anti-inflammatory cytokine playing a crucial role in wound healing. IL-10 helps regulate the immune response by deactivating monocytes and macrophages and reducing the production of pro-inflammatory cytokines, thus controlling inflammation at the wound site. Not just leukocytes, but also regulatory T-lymphocytes and various immune cells contribute to the production, creating a balance between defending against infections and repairing tissues. Research on mice indicates that a lack of IL-10 leads to an overly strong inflammatory reaction, more scar tissue, and changes in collagen placement. On the other hand, higher levels of IL-10 reduce inflammation, help maintain normal strength in tissues, and lessen scarring (8–13). It is shown to exhibit qualities that have the ability to regulate and change the immune response (14). IL-10 also has the ability to reduce inflammation by limiting the generation of cytokines by immune cells. This is another way that it can reduce inflammation. Additionally, interleukin 10 (15).

Plays a part in the enhancement of the production of antibodies by plasma cells, which in turn enables these cells to survive for a longer amount of time. Glial inflammatory responses are under control thanks to the presence of IL-10, which is expressed inside the central nervous system. It is common knowledge that interleukin-10 (IL-10) plays a vital role in the reduction of inflammation in the margin’s tissues. In addition, this particular molecule has been the anti-inflammatory cytokine that has been the subject of the most exhaustive research. Therefore, the biomarker IL-10 is commonly believed to be the epitome of an immuneo suppressive cytokine that is produced within the central nervous system (16). This is because IL-10 shows immune system suppression.

### Glucose for energy or basic materials

The body uses glucose, a basic carbohydrate, to provide energy quickly. Particularly for erythrocytes and the brain, a steady supply of glucose is necessary for energy. Glycolysis uses up the majority of glucose in a typical physiological setting, while the pentose phosphate pathway makes use of the rest (17). Roughly 30% of glucose undergoes oxidation in the liver through the pentose phosphate pathway. Cells that divide quickly use the NADPH-and ribose-dependent pentose phosphate pathway. In addition to being an essential component in many metabolic pathways, glucose is a cellular energy source. The body quickly turns glucose into glycogen, which is then stored in the muscles and liver, and this process eliminates the elevated blood glucose levels that occur after eating. The process of glycerol synthesis in adipose cells requires glucose. Glucuronic acid, an intermediate product of glucose oxidation, is involved in detoxication and the uranic acid pathways produce muco-polysaccharides. Sorbitol, which is naturally found in the eye’s lens, can also be formed by reducing it. In addition to being an energy source, glucose is a building block for neurotransmitters (18). Since the brain is unable to retain glucose, it requires a steady flow of the fuel to keep working properly (19).

### Glycemia to excess

Hyperglycemia is characterized by persistently elevated blood sugar levels. Fasting hyperglycemia and postprandial hyperglycemia are suspected when blood glucose levels surpass 90–130 mg/dL following an 8-h fast (19). When blood glucose levels rise above 180 mg/dL after eating, a condition known spostprandial hyperglycemia develops. The blood glucose level is considered to be hyperglycemic if it is between 100 and 126 milligrams per deciliter (about 5, 6, and approximately 7 mill moles per liter, as stated by the standards of the American Diabetes Association). When the glucose level in the blood is higher than 7 mmol/L, the individual is deemed to have diabetes. Hyperglycemia that is chronic, is one of the primary factors that contributes to organ damage. Hyperglycemia that is maintained for an extended period of time can have a number of detrimental consequences on many cell types, which can lead to glucose toxicity (20). As a result of a multitude of investigations (20–22), the molecular pathways that under liehyperglycemia and its repercussions have been identified. Since hyperglycemia causes reactive oxygen species (ROS) to be produced and it is possible that DNA is damaged (23, 24), it may also play a role in the development of an inflammatory response (25). Hyperglycemia has been shown to stimulate both the polyol pathway and the hexosamine pathway, according to investigations that have been conducted. The activation of protein kinase C (PKC), the formation of end products (AGEs), and advanced glycation are all elements that are involved. DNA to be damaged (23, 24), it may have a role in the development of an inflammatory response (25).

Research has demonstrated that hyperglycemia can promote both the polyol pathway and the hexosamine pathway. Advanced glycation, the creation of end products (AGEs), and the activation of protein kinase C (PKC) are some of the additional variables that may contribute to the development of hyperglycemia (26). Under conditions in which primary rat adipocytes were exposed to glucose, a decrease in insulin sensitivity was found (27). Additionally, it has been proven that insulin resistance that is produced by hyperglycemia is not easily reversible (25). The toxicity of hyperglycemia is exerted on a variety of macromolecules, including DNA and proteins, as well as organelles and cells. Hyperglycemia has also been linked to the development of microvascular and macrovascular problems in diabetic patients (28), which is a consensus that is widely well acknowledged. In the later stages of diabetes, problems such as nephropathy, retinopathy, neuropathy, atherosclerosis, and infection-prone conditions occasionally manifest themselves. In advanced diabetes, sustained high blood sugar levels can cause various serious problems like kidney disease, eye conditions, nerve damage, blood vessel issues, and a greater risk of infections. These issues mainly result from complex biological processes that occur due to long-term high glucose levels, which include oxidative stress, inflammation, and problems in metabolism. 1- Diabetic Nephropathy (kidney disease): Continuous high blood sugar levels harm kidney cells, particularly podocytes and the glomerulus, due to the presence of advanced glycation end-products (AGEs) and oxidative stress. This results in poor filtration, leakage of proteins (albuminuria), and ultimately leads to kidney failure. Additionally, problems with insulin signaling in podocytes play a role, separate from the effects of elevated blood sugar. Changes in kidney metabolism and blood vessel function are important elements in the progression of the disease (29). 2- Diabetic Retinopathy (Eye Condition): Elevated glucose levels harm the tiny blood vessels in the retina and lead to oxidative stress and inflammation. As a result, this triggers microvascular problems, reduced blood flow, and the growth of abnormal new vessels, ultimately harming both the structure and function of the retina. 3-Diabetic neuropathy, or nerve damage, occurs when high blood sugar levels cause harm to peripheral nerves and Schwann cells, resulting in the breakdown of nerve fibers. Additionally, the damaged metabolism of fats makes the nerve injury even worse. 4- Atherosclerosis Diseases of Large Vessels: in people with diabetes, the formation of plaque in arteries speeds up due to inflammation, oxidative stress, and imbalanced fat metabolism. 5- Conditions Susceptible to Infection: When blood sugar levels are consistently high, it affects how well immune cells work and slows down healing. As a result, people with diabetes are at a higher risk of getting infections, especially skin infections and diabetic foot ulcers, because of weakened immunity and inadequate blood flow (30). There are a number of additional repercussions that may be connected with this illness (31). Some of these consequences include hypothyroidism, hyperthyroidism, non-alcoholic fatty liver disease, decreased joint mobility, and fluid retention.

### Polyneuropathy caused by diabetes

**Figure 1:**
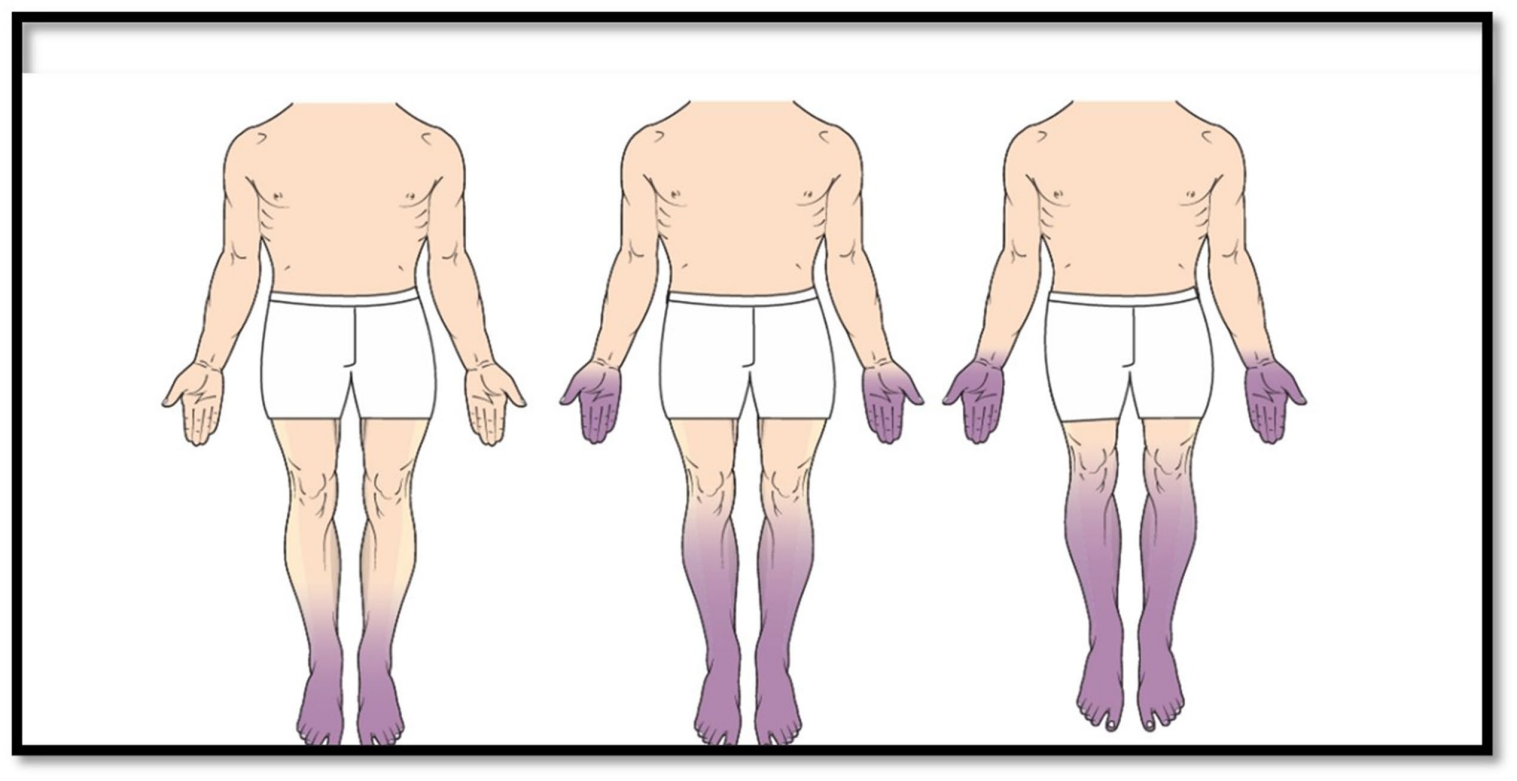
Illustration of progressive “stocking and glove” sensory changes in a patient with progressive diabetic polyneuropathy. Sensory symptoms and signs begin in the distal territories of sensory nerves in the toes before fin- gers with a gradual spread proximally. (From Zochodne D. W., Kline G., Smith E. E., et al.: Diabetic Neurology. Informa Healthcare, New York, 2010, with permission).

The most prevalent type of peripheral neuropathy is known as diabetic polyneuropathy with diabetes. The illness is present in around 50 % of diabetic people who have either type 1 or type 2 diabetes, according to a comprehensive evaluation. There is a possibility that roughly 15 % of these patients will get symptomatic polyneuropathy (32). Studies that only included hospitalized patients or studies that focused solely on clinical indications of polyneuropathy have produced prevalence numbers that are lower than those found in published research. The majority of the forms are sensory, with or without a minor degree of input from the motor system. Prickling, tingling, “pins and needles,” burning, crawling, itching, electrifying, stinging, jabbing, and tight sensations in the legs, feet, hands, and fingers are some examples of positive sensory symptoms that are frequently experienced. Other examples include tingling, prickling, and “pins and needles.” Several different sensations can be described using these sensations as a frame of reference. Warm stimuli have the potential to be misunderstood as being cold, and cold stimuli have the potential to be misunderstood as being either warm or hot. Both of these possibilities are possible. The emergence of discomfort from a range of causes is the defining characteristic of the disorder known as allodynia. Typically, harmless stimuli, is accompanied by nocturnal burning of the feet. There is a strong correlation between many of these symptoms and acute pain, which can sometimes become unmanageable. In most cases, the symptoms are symmetrical and initially limited to the toes. However, as time goes on, they expand to more proximal portions of the feet, legs, and fingers (Figure 1). As a result of the involvement of the sensory axon terminals factors that contribute to the development of foot ulcers. Early diabetic polyneuropathy is associated with a lower incidence of motor weakness; nonetheless, this weakness can later develop to distal weakness of foot and toe dorsiflexion, which puts patients at risk for falling. A waste of the intrinsic foot muscles is a symptom of weakness that is accompanied by weakness. Erectile dysfunction in men, distal loss of sweating, orthostatic symptoms such as “dizziness,” and bowel and bladder dysfunction are all examples of symptoms that are commonly associated with concomitant disorders of the autonomic nervous system. It is vital to conduct a comprehensive neurologic examination in order to evaluate diabetic polyneuropathy since this examination offers a direct evaluation at a discounted cost. Even while it is reasonable to anticipate that there would be some variation in the findings, particularly in individuals who are experiencing the disease at an extremely early stage, the examination continues to be the gold standard for diagnosis. Quantitative methods or electrophysiologic evaluation, which are considered auxiliary tests, do not replace the examination.

In the distal region of the patient’s body, there is a loss of sensitivity to light touch, pinprick, cold, and vibration with a 128-Hz tuning fork, according to an examination of the patient’s sensory capacities made by the medical professional. After obtaining anesthesia, patients who have a more severe sensory loss may not be able to discern between sharp (pinprick) and dull (analgesia) feelings, or they may not even be able to feel light touch at all. This is because they have lost the ability to perceive both types of sensations. As an additional component of the neurologic evaluation, the Femmes? Weinstein (10 g) monofilament test is a useful tool. Over the dorsum of the great toe or other specific areas of the foot, the filament is pressed against the skin until it forms a C-shaped bow for a single second. This is done during the procedure. After that, the patient is questioned about whether the stimulus is observed through the utilization of the probe. Utilizing the Ryder? Safer tuning fork is a method that can be utilized to acquire semiquantitative information concerning vibratory sensory perception. Depending on the degree of the ailment, the lack of vibration may impact on the distal toes, the foot below the ankle, or even more extensive locations. Furthermore, the condition may affect larger areas. Except for situations that are especially severe, testing for proprioceptive anomalies in the toes is typically considered to be considered normal. There is often a correlation between more severe sensory loss and distal motor wasting, such as in the extensor digitiform breves muscle, as well as accompanying weakness, notably in the region of foot and toe dorsiflexion. This is because distal motor wasting is associated with progressively more severe sensory loss. Both foot ulcers and a destructive arthropathy caused by recurrent damage, which is referred to as a Charcot joint by medical professionals, are possible outcomes for patients. Foot ulcers are more common than a Charcot joint. A typical symptom of early diabetic poly neuropathy is the loss of the muscle stretch reflex at the ankle. This can occur in a number of different ways. All of the body’s deep tendon reflexes are lost when the disorder is severe enough to cause irreversible damage. It is possible that the amount of sweating that has occurred has caused the feet to become dry. A loss of distal pulses and the appearance of femoral bruits are two symptoms that may be experienced by patients who are suffering from concurrent atherosclerosis. Persons who have involved of the autonomic nervous system are more likely to suffer postural hypotension if they experience a drop in their systolic blood pressure of more than 20 mmHg or a drop in their diastolic blood pressure of 10 mmHg (22). Consequently, it is necessary to conduct an assessment of the orthostatic vital signs. At Dyck et al. (33) and Malik et al. (34) subcategories of diabetic polyneuropathy have been formed on the basis of whether or not the ailment involves involvement of the patient’s large or microscopic fibers. These subcategories have been established using the aforementioned criteria. Persons who have large-fiber polyneu ropathy are more likely to experience a loss of sensitivity to light touch, vibration, and proprioception than other persons. As an additional complication of this ailment, individuals may also face the possibility of experiencing ataxia of gait. Those who suffer from small-fiber polyneuropathy frequently experience autonomic dysfunction, and neuropathic pain, especially throughout the night, is also a prevalent symptom of this condition. As a result of this disease, pinprick and heat sensitivity are both impaired. A number of different kinds of diabetic polyneuropathy have been linked to the presence of pain that is characterized by neuropathic pain. The 23rd for the aim of conducting clinical research, a number of different methods have been applied over the course of time in order to assess the degree of severity of diabetic polyneuropathy. The San Antonio criteria classify polyneuropathy as class I if it does not exhibit any signs or symptoms but has abnormalities on electrophysiologic testing, autonomic testing, or quantitative sensory testing (QST), 1-Clinical screening tests are basic examinations commonly performed at the bedside. They are typically done once a year to check for diabetic peripheral neuropathy (DPN). These tests involve evaluating pinprick sensation, temperature feeling, ankle reflexes, vibration awareness with a 128-Hz tuning fork, and pressure sensitivity using a 10-gram monofilament. Employing different tests together improves the ability to identify neuropathy in individuals with diabetes. 2-Nerve Conduction Studies (NCS): NCS is widely regarded as the best method for diagnosing and assessing the severity of polyneuropathy. This test evaluates the speed, strength, and delay of electrical signals in different motor and sensory nerves, giving clear, measurable insights into how nerves are functioning. Commonly examined nerves consist of median, ulnar, peroneal, tibial, radial, and sural nerves. The detection of issues in two or more nerves usually supports a diagnosis of DPN. Even in the absence of symptoms, NCS can indicate early stages of neuropathy, facilitating prompt diagnosis and tracking (35). 3- Quantitative Sensory Testing (QST) is a method that evaluates how well small and large fibers work by checking the response to different stimuli such as vibration, cold, and heat. This testing is helpful for diagnosing conditions and tracking their development over time. Among the tests conducted are vibration perception threshold (VPT) and temperature discrimination, with VPT being popular because it is safe and can be reliably repeated (9). 4- Electromyography (EMG) involves using needle EMG together with nerve conduction studies (NCS) to assess the electrical activity of muscles. This combination offers extra insights into how nerves and muscles are affecte. 5- Autonomic Function Tests: Because diabetic neuropathy may impact autonomic nerves, doctors sometimes use tests that assess heart rate variability, how blood pressure responds, and sudomotor function (36, 37). 6- Advanced Techniques: A skin biopsy that measures the density of intraepidermal nerve fibers (IENFD) provides a direct evaluation of small fiber neuropathy. It is becoming recognized as an accurate diagnostic method, particularly for neuropathies that are early-stage or mainly impact small fibers. 7- Neurological scales that are standardized evaluate the severity of symptoms and physical signs of neuropathy. They assist in determining the disease stage and tracking clinical results (38). In clinical studies, it is advised to use various evaluations such as symptoms, signs, nerve conduction studies (NCS), quantitative sensory testing (QST), and autonomic tests to assess the severity of diabetic polyneuropathy effectively. Generally, a diagnosis needs to be confirmed by pairing nerve conduction studies with symptoms or signs. The reliability and sensitivity of tests like NCS and vibration perception threshold (VPT) are helpful for tracking the progression of neuropathy in research trials.

This criterion was developed in the United States. Class II is characterized by either indications or symptoms, or both, depending on the degree to which the disease is considered severe There are twenty-four: different scales for measuring neuropathy include the Modified Toronto Neuropathy Scale, the Utah Neuropathy Scale, the Michigan Neuropathy Scale, and the Mayo Clinic diabetic polyneuropathy classification., are some of the other scales that are not discussed in this page about neuropathy (39–41).

### Syndrome of the carpal tunnel

The transverse carpal ligament compresses the median nerve of the wrist, leading to carpal tunnel syndrome. Common causes of this ailment include using thumb and wrist motions repeatedly. Tingling, soreness, and a lack of sensation in the thumb, index, and middle fingers are signs of this condition. Both waking up and going to sleep are associated with worsening of these symptoms. Asymptomatic carpal tunnel syndrome can be diagnosed in 20 to 30 percent diabetics with electrophysiologic testing (42, 43). Nevertheless, symptomatic carpal tunnel syndrome is more common. Neuropathies affecting the upper limbs can be differentiated from carpal tunnel syndrome using electrophysiologic testing that shows a selective slowing of median nerve fiber conduction across the carpal tunnel. Patients suffering from carpal tunnel syndrome may find relief by reducing their activity level and using wrist splints at night. There is only one operation available, and that is decompression, which involves severing the transverse carpal ligament and may be thought of as a curative treatment (44). It is possible that non-diabetics’ recoveries are more robust than diabetics’, especially in cases when glycemic control is inadequate. The reason being, diabetic neuropathy makes nerve regeneration more difficult. Carpal tunnel syndrome, a type of entrapment neuropathy, is more common in both diabetic and non-diabetic patients. The incident is more likely to involve the dominant hand than any other hand, and it disproportionately affects women compared to men. “Tinel sign” refers to the process of tapping on the median nerve at the wrist to bring up positive sensory symptoms comparable to the patient’s symptoms further away from the wrist. Even though the Phalen sign—which involves flexing and holding both wrists against each other for one minute to simulate tingling—could be present, it is neither sensitive nor specific. For people with mild carpal tunnel syndrome, the symptoms might not be noticeable. Later on, the area around the median nerve could experience a loss of sensation. The abductor pollicis brevis, often called the thenar muscles, weakens and atrophies in more advanced forms of carpal tunnel disorder (44).

### At the elbow, ulnar neuropathy is found

When ulnar neuropathy is present at the elbow, the patient experiences pain and sensory problems in the middle half of the ring finger and the fifth digit. These symptoms can sometimes radiate into the palm and even reach the wrist near the elbow. These fingers, together with the medial volar and dorsal hand, all the way up to the wrist, are at risk of experiencing sensory loss. Because of the possibility of wasting and weakness of the intrinsic ulnar-innervated hand muscles, particularly in the first dorsal interposed muscle, the patient may have difficulty abducting or adducting their fingers while they are experiencing this condition. When the ulnar nerve in the elbow is manipulated, it is possible for the sensation to be sent to the hand, which in turn would duplicate the symptoms. Predisposing variables include the consumption of ethanol as well as previous elbow injuries or fractures. One of the most prevalent causes of the illness is when the patient leans on their medial elbow, which causes the nerve to become compressed. It is estimated that roughly 2 % of persons who have diabetes mellitus have ulnar nerve entrapment when they are diagnosed with the condition (43). Reduced speed of ulnar motor and sensory transmission across the elbow, absence of action potentials from the ulnar sensory nerve and compound motor, and, in very unusual cases, a complete blockage of conduction across the elbow are all symptoms of this condition. Electrophysiologic study has confirmed these results. Electromyography allows one to detect denervation when applied to the afflicted muscles. You can treat ulnar neuropathy by shifting your elbow or by cushioning the area around the nerve. These two approaches work well. If the lesion is affecting motor axons, it is causing symptoms, and is getting worse despite conservative treatment, decompression may be the best option. Surgical decompression is a reasonable strategy in most circumstances, according to current clinical practice, even if there have not been any controlled clinical trials done on diabetic patients to indicate that it improves long-term outcomes.

### A peripheral neuropathy caused by diabetes

It is typical for microvascular disorders of the eyes, kidneys, and peripheral nerves to develop resulting from both type 1 and type 2 diabetes’ persistent hyperglycemia (45). Usually beginning with the small nerve fibers of the lower limbs, diabetic peripheral neuropathy (DPN). Affects Nerve loss resulting from this disorder can induce a delayed start of symptoms, including foot pain, tingling, numbness, muscular weakness, increased sensitivity to touch, and heat intolerance (46). Patients describe a sensation like that of a wooden or numb foot in either one or both feet, but first presentations of these symptoms may be modest and vague. Usually, the development of DPN is accompanied by a burning sensation marked by a “stocking and glove” distribution that gets progressively more uncomfortable during the day (47). The most often occurring sign of diabetic peripheral neuropathy (DPN) is distal symmetric limb numbness accompanied by loss of sensation. Furthermore, DPN can cause neuropathic pain in about 20 % of diabetics as well. Pruritus, hyperalgesia, and induced pain rank as the most often occurring forms of pain (48). Common forms of pain are cauterizing, electrical, and extreme ones. Apart from this, the mix of hyperglycemia and metabolic disorders damages the immune system and influences its functioning in the body. Unnoticed and cunning, this cut could develop an infection and seriously harm the limbs (49). 1-Immune System Issues and Risk of Infection: When blood sugar levels are too high (hyperglycemia) along with metabolic problems in diabetes, the immune system is negatively affected, making it less effective at battling infections. This issue can reduce healing of wounds and the overall immune response, particularly in areas like the feet and legs. As a result, small cuts or injuries can get infected without immediate signs, which might result in serious problems like ulcers and gangrene, and in some cases, even lead to amputation of limbs (50). 2-Causes of Nerve and Immune Damage: When glucose levels remain high for a long time, it harms peripheral nerves by causing oxidative stress, inflammation, problems with mitochondria, and issues with how glucose and fats are processed. Moreover, high blood sugar leads to the formation of advanced glycation end-products (AGEs), which build up in nerve tissues and worsen nerve function. This harm also disrupts the blood supply to the nerves, making it harder for immune cells to reach them (51). 3-The Function of Immune Cells in Neuropathy: recent studies show that immune cells like macrophages enter peripheral nerves as a protective measure, trying to reduce nerve damage caused by diabetic neuropathy. Despite this, the ongoing metabolic issues and constant injury can overwhelm these protective actions. As immune system problems increase, the risk of infections rises, leading to more severe injuries in the limbs (52). The most common cause of non-traumatic lower limb amputation in most countries with high earnings, according to recent studies, is distal proximal neuropathy (DPN) (53). In high-income countries, diabetic peripheral neuropathy (DPN) is the leading reason for non-traumatic amputation of the lower limbs. This condition can cause diabetic foot ulcers and infections, which may result in the need for amputation if they are not treated correctly: 1- Complications from diabetes lead to approximately 60–70% of all amputations in the lower limbs around the world. A major reason for this is diabetic peripheral neuropathy (DPN), which results in a loss of sensation. This lack of feeling can cause injuries that go unnoticed and can lead to infections, eventually resulting in ulcers and gangrene (54, 55). 2-Studies show that around 44–51% of people with diabetes experience peripheral neuropathy, and many of these individuals go on to develop foot ulcers, which heightens the likelihood of needing an amputation (56). 3- People with diabetes face a risk of needing lower limb amputations that is as much as 15 times greater than those without the condition. This increased likelihood mainly results from issues like nerve damage, inadequate blood flow, and infections (57). 4- Data indicates that amputations below the knee due to diabetes-related issues are frequent, occurring at a rate of about 9.8% in the groups examined, which underlines the impact of amputations associated with diabetic peripheral neuropathy (DPN) (56). 5- Improvements in professional foot care and the use of special shoes have lowered the number of amputations; however, diabetic peripheral neuropathy (DPN) still causes most non-traumatic amputations (58). 6- The disease process begins with nerve damage that results in loss of sensation. This means that small cuts or pressure wounds can be overlooked and become infected. In diabetes, an already weakened immune system and slow healing make this worse, leading to tissue death that may require amputation (Table 1).

**Table 1:** A summary compiling key studies and reviews that have described limb-related sensory symptoms—such as tingling, cramping, twisting, coldness, or sensory loss—in the context of hyperglycemia or diabetic neuropathy.

Author(s), Year	Study design	Population/Model	Limb-related sensory symptoms	Mechanistic insights/Biomarkers	Interventions tested
Kender et al. (101)	Clinical, observational	141 patients with type 2 diabetes	Upper & lower limb sensory loss, tingling, reduced dexterity	Sensory deficits in large/small fibers; Quantitative sensory testing	Not focus, but overall glycemic control assessed
Marshall et al. (102)	Literature review	Clinical and preclinical studies	Paresthesia, sensory loss, cramping, pain, hypoesthesia	Neurophysiological/biomarker review: evoked potentials, nerve excitability, microneurography	Not primary focus
StatPearls/NCBI (103)	Review	Clinical	Numbness, tingling, burning, weakness of limbs	Nerve fiber subtype involvement, loss of ankle reflexes	Not discussed
Tseng et al. (104)	Observational	Diabetes patients	Numbness, tingling, pain in fingers/toes	Not detailed in summary	Not detailed in summary
Chong, and Souayah (105)	Review	Clinical	Loss of feeling, tingling/burning, cramps, coldness, pain	No mechanistic focus	Not detailed
Vinik et al. (106)	Systematic Review	Clinical and experimental	Sensory impairment (numbness, tingling, loss, pain)	Focus on oxidative stress, inflammation, microRNA, demyelination	Diagnostic/prognostic biomarker focus
Zilliox and Russell (107)	Patient/clinical guide	Clinical	Tingling, numbness, burning, shooting pain, loss of temperature, coldness, loss of coordination	Not mechanistic, symptom-centered	Not focus
Fujita et al. (108)	Patient education	Clinical	Tingling, numbness, muscle cramping, limp pain/weakness, coldness	Not mechanistic, symptom-centered	Discusses glycemic control
Prashantet al (109).	Review	Clinical	Small fiber neuropathy with pain/cramping/loss	Emphasis on small vs. large fiber pathology	Gabapentin, pregabalin, duloxetine, amitriptyline, opioids, topical capsaicin
Fan et al. (110)	Review	Clinical/experimental studies	Hyperesthesia, hypoesthesia, numbness, pain, coldness	Oxidative stress, degeneration, plasma/serum biomarkers, gene polymorphisms	Focus on regenerative medicine and cell therapy

### Cellular and molecular mechanisms

#### Persistent hyperglycemia triggers several damaging processes

Glycation has been recognized for a long time as a factor in the development of diabetic neuropathy (59–61). In the nerves of diabetics, every part of nerve tissue can become overly glycated. Research has shown that advanced glycation end-products (AGEs) accumulate in both human and animal diabetic nerves across all components of peripheral nerve tissues (60, 61). These accumulations can be found in stromal collagen, the axoplasms of nerve fibers, Schwann cells, and endoneuria vessels. The amount of AGE found, as measured by carboxy methyllysine immunoreactions, was closely linked to a decrease in the density of myelinated nerve fibers (62). Therefore, AGE is believed to cause harmful effects in the endoneurium, primarily through direct toxicity to nerve tissues and contributing to endoneurial microangiopathy. In studies conducted in laboratories, Schwann cells demonstrated evidence of cell death and secreted tumor necrosis factor (TNF)-α along with other inflammatory substances when exposed to a high concentration of AGEs (63). Moreover, the glycated structures of axonal components like tubulin and neurofilaments disrupted the transport of signals along the axons, resulting in damage to the fibers far from the cell body (44). Furthermore, the glycation of collagen in the basement membrane, as well as laminin and fibronectin, also obstructed the healing processes in nerves affected by diabetes (64, 65).

The hexosamine pathway is activated by high blood sugar levels, which causes damage to Schwann cells and nerve cells through inflammation and oxidative stress, eventually leading to diabetic peripheral neuropathy (DPN). Under normal circumstances, only a small portion of fructose-6-phosphate from glycolysis enters this pathway, where it is changed into glucosamine-6-phosphate by the enzyme glutamine fructose-6-phosphate amidotransferase (66). Following this, glucosamine-6-phosphate is further converted into uridine diphosphate-N-acetylglucosamine (UDP-GlcNAc). This compound is a crucial activator for O-linked-beta-D-N-acetylglucosamine (O-GlcNAc) transferase, which adds O-GlcNAc to certain serine and threonine residues on important transcription factors such as specificity protein 1 (Sp1) (67). However, when blood sugar is high, the activity of the hexosamine pathway increases, activating the Sp1 pathway. Sp1 helps control the expression of several glucose-responsive “housekeeping” genes like plasminogen activator inhibitor-1 (PAI-1) and transforming growth factor-β (TGF-β) (68, 69). In a controlled study, it was found that when tissue fibrinogen activator was not detected, there was a four- to sixfoldincrease in peripheral nerve microvascular density in the diabetic peroneal outer membrane and intraneural vessels. This suggests that an increase in PAI-1 leads to microvascular ischemia and thrombosis in diabetic neuropathy (70). Additionally, TGF-β can cause cell death and axonal damage by producing reactive oxygen species (ROS) (71). Further investigation into the expression of various transcription factors and their associated molecules within the hexosamine pathway may pave the way for new treatment options for DPN in the future.

### Hyperglycemia-induced pathways leading to DN

- **Activation of the Polyol Pathway:** Aldose reductase transforms excess glucose into sorbitol, leading to an imbalance in cell osmotic and lowering antioxidant levels. This condition heightens the risk of nerve damage (50, 72).
- **The creation of Advanced Glycation End-products (AGEs):** When blood sugar levels are consistently high, AGEs form. These compounds change nerve proteins and lead to inflammation and oxidative harm.
- **Oxidative and Nitrosative Stress:** When neurons metabolize too much glucose, it results in an overproduction of reactive oxygen species (ROS) and reactive nitrogen species (RNS). This excess can harm mitochondria, disrupt axonal transport, and eventually lead to the death of neurons (50).
- **Microvascular Dysfunction**: High blood sugar levels damage the tiny blood vessels that nourish nerves, which restricts the supply of oxygen and nutrients, contributing to nerve degeneration and low oxygen levels in the nerves (72, 73).
- **Inflammation Triggering:** Elevated glucose levels lead to the activation of immune cells, such as microglia. This process causes the release of inflammatory cytokines and harmful substances that worsen nerve damage.

### The link among lowered glyoxalase I (Glo1) activity, the buildup of MG, and harm to nerve cells

A decrease in the activity of glyoxalase I (Glo1) causes methylglyoxal (MG) to build up. This toxic substance is mainly created as a byproduct during glycolysis. When MG levels rise, it triggers oxidative stress, harms the mitochondria, damages proteins and DNA, and finally leads to the death and damage of neurons.

- **MG toxicity and neuron sensitivity**: Neurons are particularly at risk from MG due to their limited ability to modify glycolysis when under stress, leading to increased MG levels. This substance causes the generation of reactive oxygen species (ROS), harms DNA, alters proteins, reduces ATP, and triggers cell death in neurons (74, 75).
• **Importance of Glo1:** Glo1 is a common enzyme that helps detoxify by changing MG into harmless D-lactate, using glutathione as a helper. When Glo1 works less effectively or is not produced enough, it makes MG removal less efficient, causing an accumulation of MG in nerve tissue (74, 75). This reduced detoxifying function is seen in models of brain injuries and neurodegenerative diseases.
- **Effects of high MG levels on the body**: Increased amounts of MG lead to problems with mitochondrial function and cause oxidative stress, which disrupts neuron activity. High MG levels can change the membrane potential of mitochondria, lower ATP production, and trigger cell death pathways related to oxidative harm (76). Additionally, MG encourages the creation of advanced glycation end-products (AGEs), which play a role in neurodegeneration.
- **Inflammation and signaling pathways:** The buildup of MG leads to the release of pro-inflammatory cytokines and triggers stress pathways like MAPK and ERK. These processes can worsen neuronal cell death and impair synaptic function (76).

### Flowchart: key mechanisms linking hyperglycemia to limb sensory symptoms in diabetic neuropathy

#### Chronic hyperglycemia

- Triggers multifactorial metabolic and vascular damage in peripheral nerves (77).

#### Metabolic pathways that become active due to high blood sugar levels: an increase in the flow of the polyol pathway

- Conversion of glucose to sorbitol → osmotic stress, reduction of NADPH → increased oxidative stress damaging nerves (77).

#### Advanced glycation end products (AGEs) formation

- Non-enzymatic glycation of proteins/lipids → structural nerve damage; activation of receptor for AGEs (RAGE) → inflammation and oxidative stress (78).

#### Protein kinase C (PKC) activation

- Alters nerve blood flow and vascular permeability → nerve ischemia and dysfunction (78).

#### Hexosamine pathway overactivation

- Alters gene expression (e.g., PAI-1 and TGF-β) promoting microvascular ischemia, oxidative stress, and neuronal apoptosis (78).

#### Mitochondrial dysfunction and excess reactive oxygen species (ROS)

- Increased oxidative stress damages nerve mitochondria, membranes, and DNA.

#### Cellular and molecular consequences

- Elevated oxidative stress and inflammation damage nerve cells.
- Activation of Poly (ADP-ribose) polymerase (PARP) → DNA repair depletion and worsened inflammation → neuronal dysfunction.
- Endoplasmic reticulum (ER) stress → unfolded protein response → apoptosis of neurons.
- Vascular dysfunction → impaired nerve blood flow (ischemia) worsens nerve injury.
- Impaired Na^+/K^+ ATPase pump function → altered nerve conduction velocity.

#### Nerve tissue damage

- Distal axonal degeneration (long sensory neurons most vulnerable).
- Demyelination and Schwann cell dysfunction.
- Loss of neuronal repair and support mechanisms.
- Sensory nerve fiber damage leads to an altered limb sensation (79).

## Clinical outcome: limb sensory symptoms

- Numbness, tingling (paresthesia).
- Neuropathic pain (burning, sharp, shooting pain).
- Loss of protective sensation → increased risk of injury, ulcers (80).

### The role of vitamin D supplementation in metabolic pathology and influence diabetic retinopathy risk

Numerous clinical studies have shown that vitamin D3 supplementation reduces the level of metabolic parameters such as total cholesterol, low-density lipoproteins (LDL), and triglycerides and decreases the insulin resistance indicator, homeostatic model assessment (HOMA index)—in patients with type 2 diabetes. However, it is not fully understood how vitamin D3 can reduce the risk of metabolic disorders. Recently, the vitamin D3 receptor (VDR) and vitamin D3 metabolizing enzymes have been detected in various types of cells, including pancreatic β cells and insulin-responsive cells such as adipocytes. Adipose tissue is a major storage site for vitamin D3 and an important source of adipokines and cytokines involved in systemic inflammation (81), but also.

At certain parts of the body, such as the gums (82), there are links to consider. It has been proposed that a lack of vitamin D3, which exists alongside obesity, might connect diabetes and obesity. Research has found various genetic differences in the VDR, GC, and CYP2R1 genes that relate to the levels of 25-hydroxyvitamin D3 (25(OH)D3) in the blood and a deficiency of vitamin D3 in populations from Western countries. With the rising number of overweight people in wealthy nations, obesity seems to be an important aspect among the many risk factors linked to the identified vitamin D3 deficiency (83, 84).

A meta-analysis of nine RCTs revealed a positive, although not statistically significant, trend toward a reduction in albuminuria facilitated by VD, indicating a potential role for VD in decelerating the progression of diabetic nephropathy (85). Moreover, a significant disparity in 25(OH)D levels has been consistently observed in patients suffering from painful diabetic peripheral neuropathy (86). Intervention with VD in these patients has been correlated with a marked alleviation of neuropathic symptoms (87). The meta-analysis by Derakhshanian et al. explored the association between VD status and diabetic nephropathy, as well as the impact of VD repletion (88). The meta-analysis by Chokhandre et al. focused on the supplementation of VD and its analogs in the context of chronic kidney disease (CKD), a condition intrinsically related to diabetic nephropathy (82). Given the importance of oxidative stress as well as inflammation in the pathophysiology of diabetic nephropathy, which is modulated by VD receptor activity, the meta-analysis assessed renal outcomes such as UACR, albuminuria, and estimated glomerular filtration rate (eGFR). In particular, VD analogs such as cholecalciferol, calcitriol, and paricalcitol demonstrated a significant improvement in renal function in two RCTs, possibly due to their ability to bypass the renal-dependent 1-alphahydroxylation process. However, more extensive RCTs are required to establish their efficacy and safety profile.

Li’s review further underscored that diabetic nephropathy is a leading cause of kidney failure in diabetic patients, emphasizing the pivotal role of the renin–angiotensin–aldosterone system (RAAS) in the progression of kidney damage (89). Although RAAS antagonists are a cornerstone in the treatment of diabetic nephropathy, their effectiveness is often hampered by a compensatory increase in renin levels. VD has been shown to exert a reno protective effect by down-regulating RAAS by suppressing renin expression. In fact, mice lacking the VD receptor exhibit exacerbated diabetic nephropathy, indicating that impaired RAAS regulation is a contributing factor. When combined with RAAS inhibitors, VD analogs significantly mitigate renal injury by preventing a compensatory increase in renin.

### The role of selenium supplementation in modulating diabetic neuropathy risk by influencing oxidative stress

Selenium supplementation has been explored for its possible effect on the risk of diabetic neuropathy through its impact on oxidative stress. This essential trace mineral is a crucial component of antioxidant enzymes, notably glutathione peroxidases (90, 91). Due to its antioxidant properties, selenium has attracted interest for its potential to prevent or alleviate conditions linked to increased oxidative stress, such as diabetes mellitus (92, 93). Moreover, individuals with diabetes have been found to have lower selenium levels compared to their healthy counterparts (94). Additionally, there are indications that selenium may help in lowering insulin resistance. Therefore, it appears reasonable to consider selenium supplementation as a beneficial intervention for this group. The connection among oxidative stress, the development and progression of diabetes, its associated complications, and the function of antioxidants has been extensively examined (95). However, there is a scarcity of clinical trials investigating the effects of selenium supplementation in diabetic patients. In this context, studies focusing on selenium supplementation in diabetic individuals have indicated a decrease in thiobarbituric acid-reactive substances, an index of lipid peroxidation, as well as a reduction in nuclear factor-kappa B (96).

Biomarkers related to oxidative stress are employed to spot individuals who are at an elevated risk for complications connected to metabolic syndrome and to evaluate the proper treatments for alleviating this issue. These biomarkers include molecules that indicate lipid peroxidation, as well as protein and amino acid oxidation, and DNA oxidation. Indicators of lipid peroxidation consist of thiobarbituric acid-reactive substances, malondialdehyde, 4-hydroxy-2-nonenal, and F2 iso proteins (97). The occurrence of protein oxidation is marked by substances such as protein carbonyls, advanced glycation end products (AGEs), oxidized LDL (ox-LDL), and advanced oxidation proteins (98). DNA oxidation can be measured using markers like 8-oxo-2-deoxyguanosine, 5-chlorouracil, and 5-chlorocytosine (99). Additional significant biomarkers related to metabolic syndrome include adipokines like adiponectin and leptin. Lower levels of adiponectin are associated with the onset of metabolic syndrome, whereas leptin, a hormone that plays a role in energy metabolism, shows a positive correlation with metabolic syndrome and abdominal obesity (100).

### Strength and limitation

This review skillfully combines the most recent findings on methylglyoxal (MG) as a crucial mediator that goes beyond glucose rise with the complex effects of hyperglycemia on diabetic neuropathy (DN), including its involvement in sensory loss and wound healing deficiencies. A useful tool for researchers and doctors, it distinguishes itself by integrating clinical symptoms, molecular explanations, and treatment implications in a balanced manner.

Myriads of limitations noticed through this review one of them is lack of longitudinal human diseases. Several questions remained to be answered via research actions (e.g., strict glucose regulation, studies on auto immune biomarkers in exposed population). While diabetes modern era illness and neurotoxic effects are documented, its role in auto immune disease remains unstudied, particularly regarding mechanism like molecular mimicry and chronic inflammation. Trials investigating VD supplementation did not reveal a significant effect on urine albumin-to-creatinine ratio (UACR). These findings suggest that while VD deficiency is associated with an elevated risk of nephropathy in individuals with T2DM, current clinical trials do not support a direct causal relationship.
